# Comparative Study of Two Oxidizing Agents, Chloramine T and Iodo-Gen^®^, for the Radiolabeling of β-CIT with Iodine-131: Relevance for Parkinson’s Disease

**DOI:** 10.3390/ph12010025

**Published:** 2019-02-05

**Authors:** Ana Claudia R. Durante, Danielle V. Sobral, Ana Claudia C. Miranda, Érika V. de Almeida, Leonardo L. Fuscaldi, Marycel R. F. F. de Barboza, Luciana Malavolta

**Affiliations:** 1 Department of Physiological Sciences, School of Medical Sciences, Santa Casa de Sao Paulo, Rua Cesario Mota Junior 61, Sao Paulo 01221-020, Brazil; aninhapharma30@gmail.com (A.C.R.D.); danielle_sobral@hotmail.com (D.V.S.); luciana.malavolta@gmail.com (L.M.); 2 Hospital Israelita Albert Einstein, Avenida Albert Einstein 627/701, Sao Paulo 05652-900, Brazil; ana.miranda@einstein.br (A.C.C.M.); eriquimika@yahoo.com.br (E.V.d.A.), leonardo.fuscaldi@hotmail.com (L.L.F.)

**Keywords:** Chloramine T, electrophilic radioiodination, iodine-131, Iodo-Gen^®^, oxidizing agent, β-CIT.

## Abstract

Parkinson’s disease (PD) is a neurodegenerative disease characterized by the loss of dopaminergic neurons in the *substantia nigra pars compacta*, leading to alteration of the integrity of dopaminergic transporters (DATs). In recent years, some radiopharmaceuticals have been used in the clinic to evaluate the integrity of DATs. These include tropane derivatives such as radiolabeled β-CIT and FP-CIT with iodine-123 (^123^I), and TRODAT-1 with metastable technetium-99 (^99m^Tc). Radiolabeling of β-CIT with radioactive iodine is based on electrophilic radioiodination using oxidizing agents, such as Chloramine T or Iodo-Gen^®^. For the first time, the present work performed a comparative study of the radiolabeling of β-CIT with iodine-131 (^131^I), using either Chloramine T or Iodo-Gen^®^ as oxidizing agents, in order to improve the radiolabeling process of β-CIT and to choose the most advantageous oxidizing agent to be used in nuclear medicine. Both radiolabeling methods were similar and resulted in high radiochemical yield (> 95%), with suitable ^131^I-β-CIT stability up to 72 h. Although Chloramine T is a strong oxidizing agent, it was as effective as Iodo-Gen^®^ for β-CIT radiolabeling with ^131^I, with the advantage of briefer reaction time and solubility in aqueous medium.

## 1. Introduction

Dopamine transporters (DATs) are transmembrane proteins present on the presynaptic membrane of dopaminergic neurons, playing an important role in the regulation of intensity and duration of dopaminergic transmission [1,2].

The pathophysiology of Parkinson’s disease (PD) is characterized by progressive loss of dopaminergic neurons in the *substantia nigra pars compacta* (SNpc), leading to a denervation of the nigrostriatal tract that emits its terminal projections to the putamen and caudate nucleus with significant reduction of dopamine and, consequently, loss of DAT integrity [3,4,5].

Since it is a chronic and progressive disease, it is very important to perform an early diagnosis based on clinical evaluation then confirmed by imaging techniques. The clinical relevance of DAT integrity evaluation by diagnostic imaging techniques, such as single photon emission computed tomography (SPECT) and positron emission tomography (PET), are based on the differentiation between degenerative parkinsonism and other conditions not associated with dopamine loss, such as essential tremor, drug-induced parkinsonism, vascular parkinsonism, and psychogenic parkinsonism [6,7].

DAT-SPECT radiopharmaceuticals available for clinical use are all tropane derivatives: (i) β-CIT (2β-carbomethoxy-3β-(4-iodophenyl)tropane) and FP-CIT (N-3-fluoropropyl-2β-carbomethoxy-3β-(4-iodophenyl)nortropane) radiolabeled with iodine-123 (^123^I).; (ii) TRODAT-1 ([2-[[2-[[[3-(4-chlorophenyl)-8-methyl-8-azabicyclo[3.2 0.1]oct-2-yl]methyl](2-mercaptoethyl)amino]ethyl]amino]ethanethiolato(3-)-*N2*,*N2’*,*S2*,*S2’*]oxo-[1*R*-(exo-exo)]) radiolabeled with metastable technetium-99 (^99m^Tc) [6,8,9].

β-CIT is a molecule that has been studied since the 90s [10]. Due to its binding affinity to presynaptic DATs, radioisotope-labeled β-CIT can differentiate PD from essential tremor with a sensibility of 95% and a specificity of 93% [8]. The proposed mechanism of β-CIT interaction with presynaptic DATs involves electrostatic interactions or hydrogen bonds [11]. In nuclear medicine, ^123^I-β-CIT is a diagnostic agent for SPECT imaging used both in the initial phase (in individuals with uncertain diagnosis) and in the follow-up of PD [12].

The radiolabeling of molecules, including β-CIT, with radioactive iodine (^123^I, ^124^I, ^125^I or ^131^I) is frequently based on electrophilic aromatic substitution using several oxidizing agents such as Chloramine T (N-chloro-p-toluenesulfonamide sodium salt), Iodo-Gen^®^ (1,3,4,6-tetrachloro-3α,6β-diphenylglycouril), lactoperoxidase, and the solid-state variants as pre-coated Iodo-Gen^®^ tubes, Iodo-Beads^®^, or Enzymobeads^®^. The most commonly used are Chloramine T and Iodo-Gen^®^, at room temperature [13,14,15,16].

Chloramine T has been used since 1962 [17]. It is a strong oxidizing agent, demanding shorter reaction periods, and is soluble in aqueous solutions [15,18]. On the other hand, Iodo-Gen^®^ is a moderate oxidizing agent, requiring longer reaction times, and is insoluble under aqueous conditions [19,20].

To the best of our knowledge, until now there have been no comparative studies between these two oxidizing agents in β-CIT radiolabeling with radioactive iodine. Therefore, the present study aims to evaluate the electrophilic radioiodination of β-CIT with iodine-131 (^131^I), due to its 8.04 d half-life, using either Chloramine T trihydrate or Iodo-Gen^®^, in order to compare the radiolabeling efficiency and compound stability.

The importance of this work is based on the improvement of the radiolabeling process of β-CIT with radioactive iodine through the choice of the most advantageous oxidizing agent. This study may result in high quality radiopharmaceuticals labeled with ^123^I for use in nuclear medicine and, consequently, higher quality SPECT images of the DATs.

## 2. Results and Discussion

**Figure 1:**
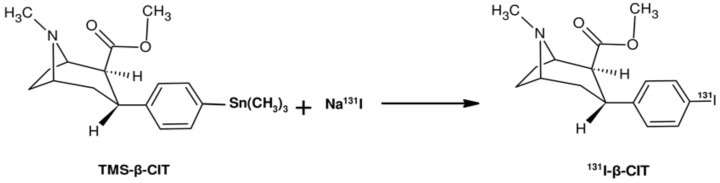
Simplified scheme of the electrophilic radioiodination of β-CIT.

The electrophilic radioiodination of β-CIT, using the precursor trimethylstannyl-β-CIT (TMS-β-CIT), was performed using either Chloramine T or Iodo-Gen^®^ as an oxidizing agent (Figure 1). The final product, ^131^I-β-CIT, was purified by solid-phase extraction (SPE) using Sep-Pak^®^ C_18_. Radiochemical purity was evaluated by both ascendant chromatography and reversed phase-high performance liquid chromatography (RP-HPLC).

**Figure 2:**
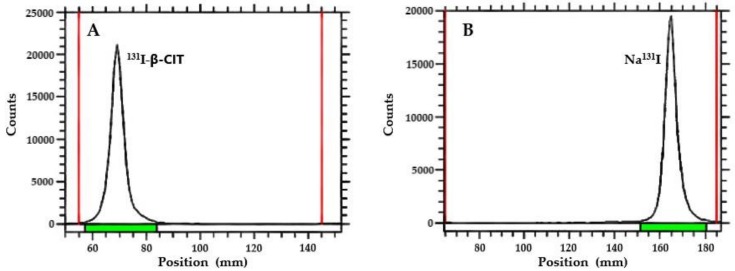
TLC-chromatograms: (**A**) ^131^I-β-CIT (R_f_ = 0.1−0.3) and (**B**) Na^131^I (R_f_ = 0.9−1.0).

Ascendant chromatography was performed on thin-layer chromatography on silica gel (TLC-SG; Al) strips using 95% acetonitrile (ACN) as an eluent (Figure 2). Results are summarized in Table 1. In this chromatographic system, Na^131^I migrates with the solvent front (R_f_ = 0.9−1.0) and ^131^I-β-CIT remains in the origin (R_f_ = 0.1−0.3). Radiochemical yield was over 95% for both radiolabeling methods. SPE purification did not significantly improve the radiochemical purity; however, this procedure is recommended in order to remove iodate ions (IO_3_^−^), which are not detected by this chromatographic system. In general, radiopharmaceuticals must present high radiochemical purity level (>90%). Therefore, this comparative study showed that both radiolabeling procedures, using either Chloramine T or Iodo-Gen^®^ as an oxidizing agent, yielded ^131^I-β-CIT with suitable radiochemical features, and that no significant differences were observed between radiolabeling methods (Table 1).

**Figure 3:**
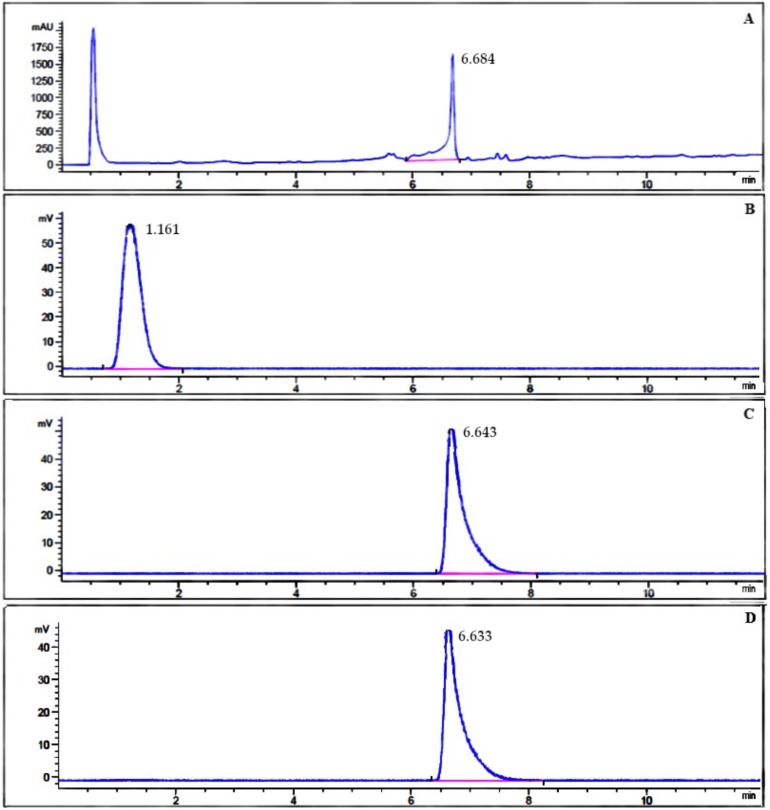
RP-HPLC chromatograms of (A) unlabeled precursor TMS-β-CIT, (B) Na131I, and (C, D) 131I-β-CIT obtained by both radiolabeling methods.

The radiochemical purity of ^131^I-β-CIT was also evaluated by RP-HPLC analysis (Figure 3) in order to confirm ascendant chromatographic data. The unlabeled precursor TMS-β-CIT was analyzed and presented a retention time (RT) of 6.68 min (Figure 3A). Free Na^131^I was also evaluated, showing a different chromatographic profile (RT = 1.16 min) when compared to unlabeled precursor TMS-β-CIT (Figure 3B). The final product, ^131^I-β-CIT, obtained by both radiolabeling methods, using either Chloramine T or Iodo-Gen^®^ as an oxidizing agent, showed high radiochemical purity (Figure 3C,D). Therefore, RP-HPLC analyses were in accordance with TLC-SG data, confirming that both radiolabeling methods yield ^131^I-β-CIT with high radiochemical purity and similar chromatographic profiles.

**Figure 4:**
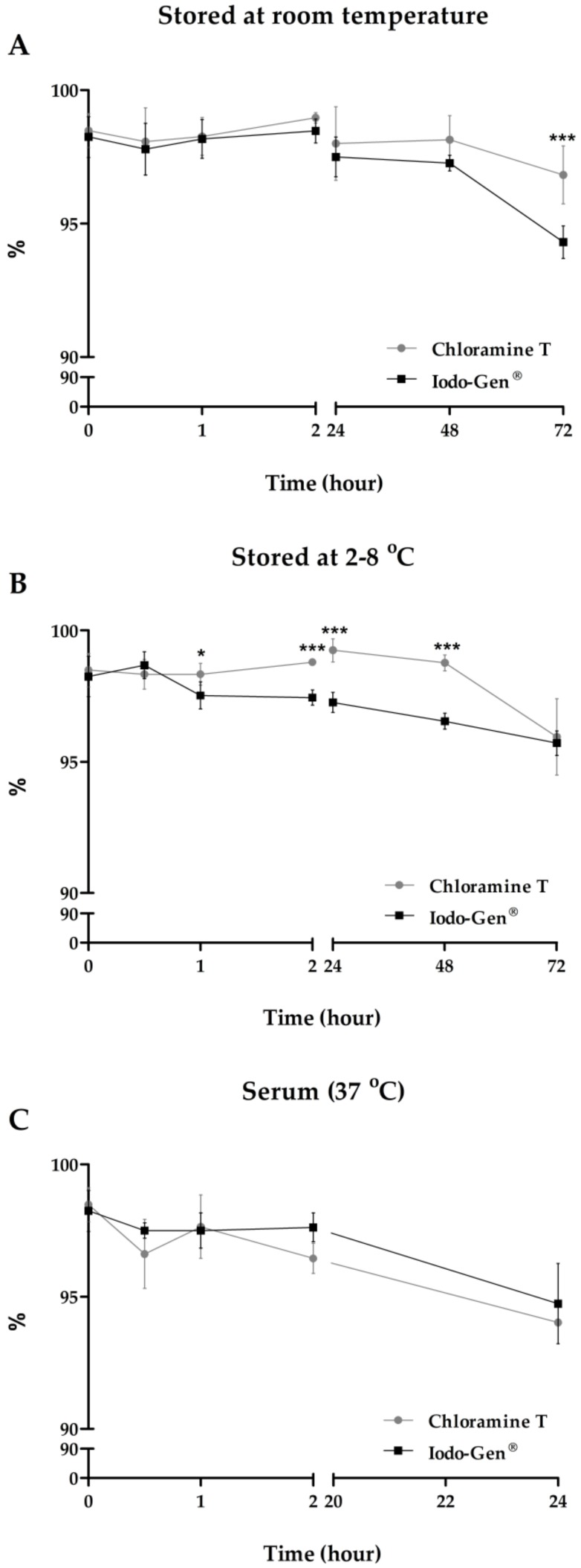
Evaluation of ^131^I-β-CIT stability. Values are expressed as mean ± SD [(**A**) and (**B**): *n* = 9; (**C**): *n* = 3]. Asterisks indicate significant differences (*p < 0.05; ***p < 0.001).

The stability of ^131^I-β-CIT was analyzed by ascendant chromatography (Figure 4). The final product was stable up to 72 h. Although slight statistical differences in the stability were observed between both radiolabeling methods, radiochemical purity maintained over 94%, regardless of the storage way, at room temperature or at 2−8 °C (Figure 4A, B). It is important to consider that these small variations in stability in reaction medium should not affect the application of ^131^I-β-CIT. Moreover, ^131^I-β-CIT serum stability was also evaluated, and data showed high stability (>94%) up to 24 h, with no significant statistical differences between the radiolabeling methods (Figure 4C).

The partition coefficient (P) of ^131^I-β-CIT was determined at room temperature by the ratio between *n*-octanol and 0.9% NaCl. For both radioiodination processes, data showed P tending to the hydrophobic range (Table 2). The hydrophobicity of ^131^I-β-CIT was also evaluated by the determination of the percentage of serum protein binding (SPB), incubating ^131^I-β-CIT with serum at 37 °C for 30 min. The results showed approximately 45% of SPB in both cases (Table 2).

The physicochemical features of the final product are in agreement, once P tending to the hydrophobic range relates to high SPB [21]. Furthermore, these data are consistent with the intended ^131^I-β-CIT clinical application, as a radiotracer for measuring presynaptic DAT density in order to provide information on the integrity of these terminals [12]. Therefore, ^131^I-β-CIT must cross the blood−brain barrier, which is easier for lipophilic molecules. In addition, it has been reported that 45−85% of SPB is related to higher uptake by the brain [22].

## 3. Materials and Methods

### 3.1. Electrophilic Radioiodination of β-CIT and SPE Purification

The electrophilic radioiodination of β-CIT was performed using either Chloramine T or Iodo-Gen^®^ as an oxidizing agent followed by SPE purification according to previous methods [14,15,23,24,25], with some modifications. The precursor TMS-β-CIT was purchased from ABX Advanced Biochemical Compounds GmbH (Radeberg, Germany).

For the Chloramine T method, an aliquot of Na^131^I solution (7.4−14.8 MBq) was added to a vial containing the precursor TMS-β-CIT (0.12 µmol/50 µL EtOH). Next, 10 µL of Chloramine T trihydrate solution (1.5 mg^.^mL^−1^) and 4 µL of HCl solution (0.1 mol^.^L^−1^) were added. The mixture (pH = 3.0−3.5) was kept at room temperature for 3 min and the reaction was quenched with 40 µL of NaOH solution (0.01 mol^.^L^−1^). The final pH was 6.0−6.5.

For the Iodo-Gen^®^ method, an aliquot of Na^131^I solution (37.0−44.4 MBq) was added to a vial containing the precursor TMS-β-CIT (0.12 µmol/50 µL EtOH). Next, 37.5 µL of Iodo-Gen^®^ solution (6.2 mg^.^mL^−1^) and 75 µL of H_3_PO_4_ solution (0.1 mol^.^L^−^) were added. The mixture (pH = 3.0−3.5) was kept at room temperature for 15 min and the reaction was quenched with 20 µL of NaOH solution (0.1 mol^.^L^−1^). The final pH was 6.0−6.5.

After each radiolabeling method, the final product (^131^I-β-CIT) was purified by SPE using Sep-Pak^®^ C_18_, preconditioned with EtOH (5 mL) and 0.9% NaCl (5 mL). Free ^131^I was removed in 0.9% NaCl (5 mL) and ^131^I-β-CIT was eluted by EtOH (2 mL).

### 3.2. Radiochemical Purity Analysis

Radiochemical purity was evaluated by ascendant chromatography (*n* = 9) and RP-HPLC.

Ascendant chromatography was performed by TLC-SG (Al) strips (Merck) using 95% ACN as an eluent. The radioactivity was determined with an AR-2000 radio-TLC Imaging Scanner (Eckert & Ziegler, Germany).

RP-HPLC analyses were performed on a 1290 Infinity II UHPLC system (Agilent Technologies, Santa Clara, CA, USA) equipped with a radioactivity detector (Eckert & Ziegler, Germany) and Open Lab ECM data system (Agilent Technologies, Santa Clara, CA, USA). The analytical column was a Phenomenex Kinetex^®^ Reversed Phase C_18_ (100 mm × 3 mm; 2.6 µm) maintained at 30 °C. Mobile phase A was 0.1% (*v*:*v*) TFA in water. Mobile phase B was 0.1% (v:v) TFA in MeOH. The gradient of mobile phase B was: 10% (0.0−1.0 min); 10−90% (1.0−8.0 min); 90% (8.0−10.5 min); and 90−10% (10.5−12.0 min). The flow rate was 0.8 mL^.^min^−1^ and the UV detector was set at 284 nm.

### 3.3. Stability Studies

The stability of ^131^I-β-CIT, stored at room temperature and at 2−8 °C, was evaluated at 0.5, 1, 2, 24, 48, and 72 h (*n* = 9). Beyond that, the serum stability of ^131^I-β-CIT, incubated at 37 °C under slight agitation (500 rpm), was evaluated at 1, 2, and 24 h (*n* = 3). In both cases, the stability of ^131^I-β-CIT was analyzed by ascendant chromatography, as described in the previous section.

### 3.4. Partition Coefficient

An aliquot of purified ^131^I-β-CIT (50 µL) was added in a mixture of *n*-octanol and 0.9% NaCl (1:1) and submitted to agitation (*n* = 5). The mixture was centrifuged (825 g; 3 min). Aliquots of 100 µL of both aqueous and organic phases were collected, and their radioactivities were measured with a 2480 automatic gamma counter Wizard^2^™ 3” (PerkinElmer, Waltham, MA, USA).

### 3.5. Serum Protein Binding

An aliquot of ^131^I-β-CIT (25 µL) was added to 475 µL of serum and incubated at 37 °C for 30 min (*n* = 5). Post-incubation, serum proteins were precipitated with 500 µL of 10% trichloroacetic acid and the content was centrifuged (825 *g*; 10 min; 3x). Pellets’ and supernatants’ radioactivities were measured with a 2480 automatic gamma counter Wizard^2^™ 3” (PerkinElmer, Waltham, MA, USA).

## 4. Conclusions

In summary, the data demonstrated that both radiolabeling methods, using either Chloramine T or Iodo-Gen^®^ as an oxidizing agent, yield ^131^I-β-CIT with similar radiochemical parameters. Although Chloramine T induces harder reaction conditions when compared to Iodo-Gen^®^, the results showed that it is possible to use Chloramine T instead of Iodo-Gen^®^ without compromising the radiolabeling result and the final product stability, provided that the physicochemical parameters of each radiolabeling process are respected (pH, reaction time, temperature). Furthermore, it is important to highlight that Chloramine T is a water-soluble reagent with strong oxidizing properties requiring a briefer reaction time, simplifying the process in nuclear medicine. Therefore, this comparative study presents the possibility of alternating between Iodo-Gen^®^ and Chloramine T in the radiolabeling process of β-CIT with ^123/131^I, depending on the availability and costs of the oxidizing agents, without changing the integrity of the final product, used for SPECT imaging diagnosis of PD in nuclear medicine.

## Figures and Tables

**Table 1:** Radiolabeling yield and radiochemical purity of ^131^I-β-CIT.

Oxidizing Agents	Radiolabeling Yield	Radiochemical Purity
Chloramine T	97.40 ± 1.17	98.48 ± 0.63
Iodo-Gen^®^	97.81 ± 0.99	98.24 ± 0.76

Values are expressed as mean ± SD (*n* = 9). No significant differences were observed for ^131^I-β-CIT obtained by both radiolabeling methods (*p* > 0.05).

**Table 2:** Partition coefficient and serum protein binding of ^131^I-β-CIT.

Partition coefficient(*P*)	Chloramine T	0.12 ± 0.02
	Iodo-Gen^®^	0.13 ± 0.02
Serum protein binding (SPB)	Chloramine T	47.44 ± 1.31%
	Iodo-Gen^®^	44.99 ± 3.06%

Values are expressed as mean ± SD (*n* = 5). No significant differences were observed for ^131^I-β-CIT obtained by both radiolabeling methods (*p* > 0.05).
